# Supplementary material for: Disappearance of superconductivity due to vanishing coupling in the overdoped Bi2Sr2CaCu2O8+δ
Source: Nat Commun. 2020 Jan 29;11:569. doi: 10.1038/s41467-020-14282-4 (PMC6989516; doi:10.1038/s41467-020-14282-4)
Supplement: Supplementary file 1 — Peer Review File [file 41467_2020_14282_MOESM1_ESM.pdf]

Reviewers' comments:

Reviewer #1 (Remarks to the Author):

The manuscript of T. Valla entitled “Disappearance of Superconductivity Due to Vanishing Coupling in the Overdoped $Bi_2Sr_2CaCu_2O_{8+d}$” is presenting an interesting ARPES study of the overdoped regime of the Bi2212 cuprate system. Using ozone doping it is made possible to tune doping in-situ. This methodology was introduced by the same principle authors in a 2018 Nature Communication publications. In that previous publication, focus was on the Fermi surface topology and the scaling between Tc and the superconducting gap. In the present manuscript the topic is related to self-energy effects induced by superconductivity. The main claim is that a bosonic coupling vanishing with superconductivity has been identified. Different bosonic scenario’s are discussed and phonons are excluded. Spin-fluctuations are therefore proposed as the most plausible. This manuscript is well-written but before making a clear recommendation, following points should be clarified.

(a) The presented data clearly demonstrate self-energy effects related to superconductivity. However, it is less clear that a bosonic mode has been identified.
The link between extracting the superconducting induced self-energy and the pairing interaction is somewhat vague in the current version of the manuscript. This point should be strengthened. For example, can the presented observations be understood/ described within Eliashberg-theory?

(b) It has been demonstrated (for example – ScienceAdvances 2, e1501329) that the “self-energy” and the "pairing self-energy" are not identical functions. Thus in line with point (a), can the authors demonstrate that their interpretations are compatible with the theoretical (Eliashberg-theory) concepts for self-energy and superconductivity?

Minor comments are:

(1) It is written that the onset of spin-fluctuations in LSCO is 8 meV. No references are given. It is a detail, but literature (PRL 102, 177006 and 102, 047001 (2009)) suggest that the onset is closer to 4 meV.
(2) The method section contains a longer paragraph explaining why data on the original Bi2212 crystal (before ozone treatment) must be analyzed differently. The absence of these data already puzzles the reader while reading the main text. Therefore, it would be useful with an indication in the main text of the fact the method section explains the ozone-free data.

Reviewer #2 (Remarks to the Author):

This is the first time that data are presented on highly overdoped bilayer BISCO. The innovation is in the sample preparation: annealing in situ of cleaved samples in ozone makes it possible to oxidize BISCO farther than is possible with conventional sample preparation techniques. The ARPES results are very interesting and these deserve to be published in Nature Communications.

But I do find that the way these data are discussed in the general context of the problem is susceptible to significant improvement.

Already for quite a long time BISCO samples have been available up to an intermediate overdoping. The present work extends it up to strongly overdoped
where superconductivity is disappearing. In fact, the main outcome is that this larger doping range

confirms trends that were already discernible in the available samples. But this is meaningful: without these new data in this large
overdoping range the case would have been much less convincing.

In summary, the results are as follows. The well known self-energy kinks seen in the ARPES dispersions have disappeared completely in the normal state of the overdoped samples in the anti-nodal direction. These re-appear deep in the superconducting state but they gradually decrease in magnitude with increasing overdoping as summarized in Fig. 3D. The highlight is that the energy of the kinks do not correlate with the "conventional" expectations: either a phonon or the magnetic resonance. Why is this very interesting?

The authors start their narrative arguing that at high doping the effects of strong correlations disappear with the effect that cuprate superconductivity should become more conventional. Without spelling it out explicitly the authors mean to say that the conventional Fermi-liquid+BCS+perturbative corrections -- let's call it Eliashberg -- get into control again. Not so long ago this was a community consensus. ARPES data played here an important role: with the eyes half closed it all looks like textbook BCS.

However, during the last two years or so a number of very confusing experimental results have changed this situation drastically. In the mean time "overdoped" has also landed on the list of the manifest high Tc mysteries. There is the observation of the Keimer group of rather strong charge order. In the present context ref. 28 is especially the big deal. The superfluid density does collapse at high doping and Tc has therefore to go to zero. Surely, it was already crystal clear as well that at the same time a conventional looking BCS amplitude gap is decreasing from ARPES data in the moderately overdoped samples. To claim that "it is governed by the weakening coupling, rather than by the superfluid density" as the authors do just indicates that they are missing the point. As spelled out in e.g. the News and Views by Zaanen accompanying ref. 28, this represents a full fledged apparent paradox. It just means that one cannot rely on Eliashberg with its simple minded coupling logic. This got spectacularly confirmed through Armitage et al. by optical conductivity measurements showing directly that only a small fraction of the electrons are in the condensate. This is actually a challenge for ARPES to detect these normal electrons: it is a quite mysterious affair while it even may be that ARPES is delusional in this regard. Optical conductivity is merciless.

The big deal is that the present data actually add further evidence that unknown physics is at work in the strongly overdoped regime! The authors should re-edit the paper in such way that this is on the forefront. The good news is that this conclusion follows directly from the analysis of the authors -- they just miss the logic in the implicit theoretical assumptions underlying their analysis.

The case that the kink in the antinodal Bogoliubon dispersions is not due to phonons is utterly convincing -- unpleasant for ref. 8 but the facts speak for themselves. But then the authors continue with an equally convincing case that the magnetic resonance does not fit either.

The issue is that when Eliashberg would work the kink should be due to the magnetic resonance! This excitation is ubiquitous as the propagating spin triplet mode forming automatically in the SC gap. But on the Eliashberg level one should "sum up the bubbles" (RPA) with the implication written in stone that there should be no other spin excitations below the BCS spin gap of 2 Delta than the magnetic resonance! No such "theorem" exists in general: it is a special effect of the perturbation theory around conventional BCS.

The authors then resort to ref. 26; this is a clever and provocative story showing directly with INS that at least in overdoped 214 spin excitations different from the resonance persists all the way down to

Delta itself! The present authors demonstrate that this appears to correlate neatly with their kink (Fig. 3c, the crosses, the squares and the dots).

But this is a breach of Eliashberg logic: there should not be spin excitations at Delta! This is actually well understood and emphasized by the authors of ref 26. Thereby the main message of this paper should be that it presents evidence that this manifest non-Fermi liquid/non BCS behavior appears to persist all the way to strongly overdoped. This is exciting.

One ramification is that it is a gross over-interpretation to associate the strength of the kink (lambda) to the "pairing glue": this would be true in Eliashberg but the authors just disqualified this theory. We don't know how pairing works in non-Eliashberg superconductivity and it is a bit painful how this lack of understanding of the theory leads to such naive over-interpretations. Surely the strength of the kink as function of doping is interesting but please refrain from reference to the superconductivity itself.

The only weakness of the present case is that there are no data available showing how the spin excitations evolve at very high dopings (no crosses in Fig. 3c above p= 0.24). I perceive this however as a reasonable extrapolation of the available data and it may be presented as a challenge for the INS community. I have actually in this regard a simple request. I perceive the spin excitations at Delta as a novelty highlighted for the first time in ref. 26 dealing with 214. I actually tried to locate the "crosses" of Fig 3c in the INS literature of BISCO (ref's 21-25) but all I seemed to find is resonance-resonance-resonance. It would be highly appreciated when the authors could include a table specifying where one can find the data showing this spin gap.

In summary, I have the guts feeling that the authors will like this report: invoking some extra theoretical discipline the data become just more interesting. The moral is that experiment is important not because of fitting the data to 60 year old theories but instead because of its power to demonstrate that the established paradigm is failing.

Reviewer #3 (Remarks to the Author):

The manuscript presents study of the boson mode in cuprates in very high doping regime. Authors demonstrate that the disappearance of the superconductivity at the overdoped side of the dome is accompanied by vanishing interaction with the boson mode. The origin and role of the boson mode was debated for several decades. There was some evidence that it is linked to high temperature superconductivity, however the situation was far from definitive. This manuscript provides substantial evidence of such link. Authors were able to measure samples in heavily doped regime, that was previously poorly or unexplored. They provided convincing evidence that the presence of the mode is linked to high temperature superconductivity in cuprates. The manuscript is well written and data presentation and conclusions very clear. The authors may want to consider citing related work: PRB 95, 174501 (2017) by Mou and coauthors.

Reviewers' comments:

Reviewer #1 (Remarks to the Author):

The manuscript of T. Valla entitled "Disappearance of Superconductivity Due to Vanishing Coupling in the Overdoped Bi2Sr2CaCu2O8+d" is presenting an interesting ARPES study of the overdoped regime of the Bi2212 cuprate system. Using ozone doping it is made possible to tune doping in-situ. This methodology was introduced by the same principle authors in a 2018 Nature Communication publications. In that previous publication, focus was on the Fermi surface topology and the scaling between Tc and the superconducting gap. In the present manuscript the topic is related to self-energy effects induced by superconductivity. The main claim is that a bosonic coupling vanishing with superconductivity has been identified. Different bosonic scenario's are discussed and phonons are excluded. Spin-fluctuations are therefore proposed as the most plausible. This manuscript is well-written but before making a clear recommendation, following points should be clarified.

(a) The presented data clearly demonstrate self-energy effects related to superconductivity. However, it is less clear that a bosonic mode has been identified. The link between extracting the superconducting induced self-energy and the pairing interaction is somewhat vague in the current version of the manuscript. This point should be strengthened. For example, can the presented observations be understood/ described within Eliashberg-theory?

We thank to the reviewer for the positive review and for raising very constructive points. The observed kink is related to the low-energy part of the SF spectrum that changes drastically upon transition between the superconducting and normal states and with doping. The total self-energy (both pairing and QP), should include the high energy region and should be related to the entire spin-fluctuation spectrum. As the high energy part of self-energy is not considered in the current study (our analysis was limited to 0>E>-0.1eV), we are not in a best position to perform the Eliashberg-type analysis too literally. However, what has become clear in this study is that the electronic quasiparticles and their coherence are most dramatically influenced by some bosonic excitations whose spectrum changes drastically between the superconducting and the normal states and which correlates in its strength and energy scale with superconductivity. This indicates that the bosonic excitation is in itself electron-derived. We note that the peak in the low-energy part of self-energy does indicate the energy of the involved mode. As described in the manuscript, that energy, in addition to other attributes (T-, k- and doping dependence) helped in eliminating some of the "usual" candidates and identified the most probable excitation. At the moment, our results point to the low-energy part of the spin-fluctuation spectrum as the best candidate.
Central to the suggestion that antiferromagnetic spin-fluctuations provide the pairing interaction is the idea that there is a significant coupling between the spins and the doped holes. A direct consequence is that when the d-wave gap opens in the quasiparticle spectrum, the spin-fluctuation spectrum will also be altered. Below Tc, the spin-fluctuation

spectral weight is removed from low frequencies and shifted to energies above ~2Delta, enhancing the pairing interaction.

(b) It has been demonstrated (for example – Science Advances 2, e1501329) that the "self-energy" and the "pairing self-energy" are not identical functions. Thus in line with point (a), can the authors demonstrate that their interpretations are compatible with the theoretical (Eliashberg-theory) concepts for self-energy and superconductivity?

The analysis done in the present manuscript has been focused on the "normal self-energy". We are certainly aware of the more involved analysis from the noted reference and it, in principle, could be done in our case. However, due to the limited scope of the present work, we think that such analysis would not result in a significantly more informative story. We certainly agree that the trends and conclusions already demonstrated by Bok *et al* are correct and could be expanded to the very overdoped regime studied here. We have now added a brief note on that in the manuscript. However, even though our results seem to qualitatively validate an Eliashberg-type analysis, we think that, at this point, taking it too literally would be an unnecessary overreach (as noted by referee #2).

Minor comments are:

(1) It is written that the onset of spin-fluctuations in LSCO is 8 meV. No references are given. It is a detail, but literature (PRL 102, 177006 and 102, 047001 (2009)) suggest that the onset is closer to 4 meV.

We thank to the referee for pointing to the references. Our number came from ref [28] (in the new version) where the authors define the spin gap in a slightly different way than what has been done in previous studies: they take the magnetic signal in the superconducting and normal states and define the spin gap as the energy where the difference between the two spectra crosses zero. The other way to define the spin gap, also used in the noted references, would be to define it as the energy below which the magnetic signal in the SC state is zero. We have added one older reference [40] that defines the spin gap in LSCO in accord with the latter convention and stated that the spin gap in LSCO is <8meV. We note that the lack of coherent antinodal quasiparticles in LSCO is more in line with the lower energy onset of spin-fluctuations. Such onset effectively dampens the antinodal QP and any fine structure in its dispersion.

(2) The method section contains a longer paragraph explaining why data on the original Bi2212 crystal (before ozone treatment) must be analyzed differently. The absence of these data already puzzles the reader while reading the main text. Therefore, it would be useful with an indication in the main text of the fact the method section explains the ozone-free data.

The referee is correct. We have now indicated in the main text (and added a reference in the figure caption) that the analysis of the as-grown sample has been done in the methods section.

Reviewer #2 (Remarks to the Author):

This is the first time that data are presented on highly overdoped bilayer BISCO. The innovation is in the sample preparation: annealing in situ of cleaved samples in ozone makes it possible to oxidize BISCO farther than is possible with conventional sample preparation techniques. The ARPES results are very interesting and these deserve to be published in Nature Communications.

But I do find that the way these data are discussed in the general context of the problem is susceptible to significant improvement.

Already for quite a long time BISCO samples have been available up to an intermediate overdoping. The present work extends it up to strongly overdoped where superconductivity is disappearing. In fact, the main outcome is that this larger doping range confirms trends that were already discernible in the available samples. But this is meaningful: without these new data in this large overdoping range the case would have been much less convincing.

In summary, the results are as follows. The well known self-energy kinks seen in the ARPES dispersions have disappeared completely in the normal state of the overdoped samples in the anti-nodal direction. These re-appear deep in the superconducting state but they gradually decrease in magnitude with increasing overdoping as summarized in Fig. 3D. The highlight is that the energy of the kinks do not correlate with the "conventional" expectations: either a phonon or the magnetic resonance. Why is this very interesting?

The authors start their narrative arguing that at high doping the effects of strong correlations disappear with the effect that cuprate superconductivity should become more conventional. Without spelling it out explicitly the authors mean to say that the conventional Fermi-liquid+BCS+perturbative corrections -- let's call it Eliashberg -- get into control again. Not so long ago this was a community consensus. ARPES data played here an important role: with the eyes half closed it all looks like textbook BCS.

However, during the last two years or so a number of very confusing experimental results have changed this situation drastically. In the mean time "overdoped" has also landed on the list of the manifest high Tc mysteries. There is the observation of the Keimer group of rather strong charge order. In the present context ref. 28 is especially the big deal. The superfluid density does collapse at high doping and Tc has therefore to go to zero. Surely, it was already crystal clear as well that at the same time a conventional looking BCS amplitude gap is decreasing from ARPES data in the moderately overdoped samples. To claim that "it is governed by the weakening coupling, rather than by the superfluid density" as the authors do just indicates that they are missing the point. As spelled out in e.g. the News and Views by Zaanen accompanying ref. 28, this represents a full fledged apparent paradox. It just means that one cannot rely on Eliashberg with its simple minded coupling logic. This got spectacularly confirmed through Armitage et al. by optical conductivity measurements showing directly that only a small fraction of the electrons are in the condensate. This is actually a challenge for ARPES to detect these normal electrons: it is a

quite mysterious affair while it even may be that ARPES is delusional in this regard. Optical conductivity is merciless.

We think that LSCO films might indeed be somewhat different. Our own ARPES studies have indicated that those films are much more disordered than Bi2212 material studied here (ref [37] in the old version, [42] in the revised version). The states in LSCO films are much broader and the superconductivity is essentially undetectable in photoemission spectroscopy. This is in a good agreement with the recent Raman studies on LSCO films grown by the same method (Kim *et al*, Phys. Rev. Materials 1, 054801 (2017)). It may well be that the superfluid density scales with Tc (as the superfluid volume gets reduced with the Sr doping) in these films. Indeed, there are theoretical studies showing that the both the penetration depth and the optical data on the overdoped LSCO films could be interpreted in a different way -- by applying the dirty *d*-wave BCS theory with weak Born-limit scatterers that reduce superfluid density without displaying quadratic behavior in T-dependence of the penetration depth (Lee-Hone *et al*, Phys. Rev. B 96, 024501 (2017), Phys. Rev. B 98, 054506 (2018)). We have now edited the relevant sentence in the manuscript to make an explicit distinction between the two materials. Furthermore, we do not agree with the referee's statement that ARPES somehow misses the normal electrons, whereas optics sees those. ARPES should detect such normal electrons easily, if they existed. For example, the spectrum shown in Fig 1b clearly represents a non-superconducting sample, with no condensate weight (no gap at any k). Fig. 1c, however, shows a spectrum of a superconducting sample with a very clean *d*-wave gap where the gapless excitations only exist near the node. If there were a significant normal component within the probed region, as the optical studies on LSCO films suggest, a significant non-zero intensity would exist everywhere on the Fermi surface, including the antinodes. Its weight would then have been comparable to that obtained in optical studies -- dominating over the condensate (gapped state) in most cases. This is certainly in contradiction to the ARPES observations on the overdoped Bi2212 samples.
In regard to the observation of a charge order in the overdoped cuprates – we are not sure what study the referee is referring to. If it is the work by Peng *et al*, Nature Mat. 17, 697 (2018), we would like to point out that that study was done on Pb-doped Bi2201, a material with Tcmax<30K that does not necessarily represent a universal model for all the overdoped cuprates. More importantly, we note that a charge order observed in that study does not have any temperature dependence, does not correlate with the Fermi surface (no nesting), does not produce and replicas and does not induce any gap in the electronic spectrum (Fig.1f-h from that reference). Those results could as well be an indication of some structural motif in this material structure of which is very complex even without Pb doping. However, the fact that no trace of a replicated electronic structure (shifted by Q_{co} relative to the original) was detected indicates that the "charge order" peak might not be elastic, but some soft finite energy mode. The authors of that study actually left that possibility open. Finally, we think that the mere existence of a charge order does not necessarily imply that the superconductivity has to be unconventional – an obvious example is the conventional superconductivity in transition metal dichalcogenides ($NbSe_2$) that also host CDW.

The big deal is that the present data actually add further evidence that unknown physics is

at work in the strongly overdoped regime! The authors should re-edit the paper in such way that this is on the forefront. The good news is that this conclusion follows directly from the analysis of the authors -- they just miss the logic in the implicit theoretical assumptions underlying their analysis.

The case that the kink in the antinodal Bogoliubon dispersions is not due to phonons is utterly convincing -- unpleasant for ref. 8 but the facts speak for themselves. But then the authors continue with an equally convincing case that the magnetic resonance does not fit either.

The issue is that when Eliashberg would work the kink should be due to the magnetic resonance! This excitation is ubiquitous as the propagating spin triplet mode forming automatically in the SC gap. But on the Eliashberg level one should "sum up the bubbles" (RPA) with the implication written in stone that there should be no other spin excitations below the BCS spin gap of 2 Delta than the magnetic resonance! No such "theorem" exists in general: it is a special effect of the perturbation theory around conventional BCS.

Our results indicate that superconductivity in the overdoped cuprates does not fit into the weak coupling BCS scenario as the ratio 2Delta/k_BT_c>4.2 and labmbda>1 for any of superconducting samples studied here. Only within the weak coupling spin-fluctuation model would the kink be at 3Delta. What we have found instead is that the kink (the peak position in ReSigma) remains close to 2Delta, and the ratio 2Delta/k_BT_c is larger than 5 even for the most overdoped, but superconducting sample. We note that within the spin fluctuation model, the spin excitations do exist below 2Delta – the stronger the coupling, the more reduced the energy of the spin-exciton becomes. For the non-interacting case, the energy would be at 2Delta (producing the kink at 2Delta, measured from Delta, or 3 Delta, measured from the Fermi level). As the coupling strengthens, the spin-exciton could be pulled close to Delta (as shown in references below). We note that even in the overdoped regime, the coupling is still very strong (labmbda>1) and 2Delta/ k_BT_c >4.2, pointing to strong coupling regime, where the spin fluctuation spectrum with the commensurate resonance gets renormalized. For our superconducting samples, 2Delta/ k_BT_c ranges from 5.2 (Tc=38K) to 9.2 (Tc=91K). It might not be statistically significant, but the experimental trend is that the kink energy is closer to 2Delta for the most overdoped superconducting sample (weakest coupling) and closer to Delta for the as grown sample (strongest coupling), in accord with the theoretical models.
Chubukov et al, Phys. Rev. B 70, 174505 (2004).
Abanov et al, Phys. Rev. Lett. 83, 1652 (1999).
Brinckmann et al, Phys. Rev. Lett. 82, 2915 (1999),

The authors then resort to ref. 26; this is a clever and provocative story showing directly with INS that at least in overdoped 214 spin excitations different from the resonance persists all the way down to Delta itself! The present authors demonstrate that this appears to correlate neatly with their kink (Fig. 3c, the crosses, the squares and the dots).

But this is a breach of Eliashberg logic: there should not be spin excitations at Delta! This is actually well understood and emphasized by the authors of ref 26. Thereby the main

message of this paper should be that it presents evidence that this manifest non-Fermi liquid/non BCS behavior appears to persist all the way to strongly overdoped. This is exciting.

One ramification is that it is a gross over-interpretation to associate the strength of the kink (lambda) to the "pairing glue": this would be true in Eliashberg but the authors just disqualified this theory. We don't know how pairing works in non-Eliashberg superconductivity and it is a bit painful how this lack of understanding of the theory leads to such naive over-interpretations. Surely the strength of the kink as function of doping is interesting but please refrain from reference to the superconductivity itself.

The only weakness of the present case is that there are no data available showing how the spin excitations evolve at very high dopings (no crosses in Fig. 3c above p= 0.24). I perceive this however as a reasonable extrapolation of the available data and it may be presented as a challenge for the INS community. I have actually in this regard a simple request. I perceive the spin excitations at Delta as a novelty highlighted for the first time in ref. 26 dealing with 214. I actually tried to locate the "crosses" of Fig 3c in the INS literature of BISCO (ref's 21-25) but all I seemed to find is resonance-resonance-resonance. It would be highly appreciated when the authors could include a table specifying where one can find the data showing this spin gap.

We acknowledge that our spin gap values came from ref [28] (new version). The authors of that study define the spin gap as the energy where the difference between the magnetic signals in the superconducting and normal states crosses zero. This is slightly different than the earlier convention, applied typically in LSCO, where the spin gap was defined as the energy below which the magnetic signal in the SC state is essentially zero (see our reply to Referee #1). For Bi2212, published studies (refs [23-27], new version) regularly present the difference in the spectra between the SC and the normal states, and the zero crossing (spin gap) can be directly read from those. We do not make statements about which definition is right, but the energies obtained that way are certainly somewhat less than the resonance energies and are more in agreement with our kink positions. As we note in the manuscript, the difference between these energy scales is particularly large in LSCO, where the spin gap is a small fraction of the resonance mode (<8 and 46 meV, respectively). The small spin gap in LSCO has prevented the observation (and the existence) of coherent Bogoliubov's QPs in LSCO very effectively. If the resonance mode were the scattering source, the quasiparticles would have had a much larger energy window where they could remain coherent. In order to make the distinction between the different excitations and energy scales clearer, we have now plotted different quantities in Fig. 3c in different colors.

In summary, I have the guts feeling that the authors will like this report: invoking some extra theoretical discipline the data become just more interesting. The moral is that experiment is important not because of fitting the data to 60 year old theories but instead because of its power to demonstrate that the established paradigm is failing.

We indeed appreciate referee's comments and very nice words about our experimental findings. We also agree with his view that trying to fit these findings to Eliashberg model would probably be an overreach (also see our reply to Referee #1). However, we urge the referee to find the place in the manuscript where we are trying to "fit our data to 60-year-old theories". We only claim that the coupling of antinodal electrons to "something" correlates strongly with superconductivity (both the energy and the strength of the coupling). We only suggest that the onset of AF excitations at the spin gap represents a good candidate that satisfies energy-momentum and temperature considerations. We do not attempt to use Eliashberg theory to calculate Tc. We do not insist that our excitation is the pairing glue. The theoretical work (ref [35] and Dahm et al, Phys. Rev. B 97, 184504 (2018), or [38-39] in the revised version) suggests that the most significant contribution to the pairing interaction comes from the energy above $4k_BT_c$, while the low energy spin fluctuations act as pairbreaking. Although this would imply that the mode we observe is in the range which contributes to pairing, we do not want to make an explicit statement on this in the manuscript. We think that it would be more appropriate if the theorists take our experimental results and solve the pairing model for themselves.

Reviewer #3 (Remarks to the Author):

The manuscript presents study of the boson mode in cuprates in very high doping regime. Authors demonstrate that the disappearance of the superconductivity at the overdoped side of the dome is accompanied by vanishing interaction with the boson mode. The origin and role of the boson mode was debated for several decades. There was some evidence that it is linked to high temperature superconductivity, however the situation was far from definitive. This manuscript provides substantial evidence of such link. Authors were able to measure samples in heavily doped regime, that was previously poorly or unexplored. They provided convincing evidence that the presence of the mode is linked to high temperature superconductivity in cuprates. The manuscript is well written and data presentation and conclusions very clear. The authors may want to consider citing related work: PRB 95, 174501 (2017) by Mou and coauthors.

We thank to the referee for a very positive report. We have now included the noted reference as it additionally reinforces our findings at the near-optimal doping level.

REVIEWERS' COMMENTS:

Reviewer #1 (Remarks to the Author):

The authors have replied to all comments and suggestions from the referees. Generally the impression is that the referee reports had marginal influence on the manuscript. Nevertheless, I don't mind this work being published in Nature Communication.

Regarding the discussion of spin gap in the spin excitation spectrum of LSCO. The authors decided to stick to their 8 meV energy scale and cite an older work (ref 40). Inspection of that first report of a clean observation of a spin gap, however reveals data taken up to 4.5 meV only. It is completely fair to cite early experimental results. In this case, however, it would be appropriate to cite also experiments that actually covers the excitation spectrum in the mentioned interval 0 - 8 meV.

Reviewer #2 (Remarks to the Author):

This is ironic - perhaps predictably the authors completely missed the case I was presenting aimed at turning the manuscript into a real interesting story. Their response is typical. They belong to the spin-fluctuation superglue school of thought. Whatever it takes, they assume it to be fact that BCS/Eliasbergh is a universal theory and one has only to identify the glue boson, and when this is in the form of spin fluctuations the problem is solved. But the thing to prove is whether one can depart from Eliashberg. The theoretical work quoted in their rebuttal actually departs implicitly from this assertion while there is actually no evidence whatsoever either mathematically nor experimental that Eliashbergh logic is credible. The authors even do not realise that one needs Eliashbergh in order to relate the energy scales they indeed do measure to pairing and so forth! Furthermore, they are apparently in the grib of their BCS mindset to such a degree that they dismiss all the recent indications that the overdoped state is actually really interesting. Among others, they do not seem to be aware that the Born scatterers of Hirschfeld have been shot down thoroughly by Mahmood et al recently in the context of the low superfluid density. Whatsoever, I find it a waste of my time to further attempt to help the authors to see the light. It is a bit unfortunate that the paper is to a degree polluted by theoretical biasses, However, the data are interesting and surely worthy to be published in Nature communications.

Reviewer #1 (Remarks to the Author):

The authors have replied to all comments and suggestions from the referees. Generally the impression is that the referee reports had marginal influence on the manuscript. Nevertheless, I don't mind this work being published in Nature Communication.

Regarding the discussion of spin gap in the spin excitation spectrum of LSCO. The authors decided to stick to their 8 meV energy scale and cite an older work (ref 40). Inspection of that first report of a clean observation of a spin gap, however reveals data taken up to 4.5 meV only. It is completely fair to cite early experimental results. In this case, however, it would be appropriate to cite also experiments that actually covers the excitation spectrum in the mentioned interval 0 - 8 meV.

We thank to the referee for an honest review and valuable suggestions. We note that we have chosen the spin-gap values that were obtained in the same way for all the compounds to be as consistent as possible. At the end we also decided to cite one of the references (ref [41] in the revised version) that referee recommends.

Reviewer #2 (Remarks to the Author):

This is ironic - perhaps predictably the authors completely missed the case I was presenting aimed at turning the manuscript into a real interesting story. Their response is typical. They belong to the spin-fluctuation superglue school of thought. Whatever it takes, they assume it to be fact that BCS/Eliasbergh is a universal theory and one has only to identify the glue boson, and when this is in the form of spin fluctuations the problem is solved. But the thing to prove is whether one can depart from Eliashberg. The theoretical work quoted in their rebuttal actually departs implicitly from this assertion while there is actually no evidence whatsoever either mathematically nor experimental that Eliashbergh logic is credible. The authors even do not realise that one needs Eliashbergh in order to relate the energy scales they indeed do measure to pairing and so forth! Furthermore, they are apparently in the grib of their BCS mindset to such a degree that they dismiss all the
recent indications that the overdoped state is actually really interesting. Among others, they do not seem to be aware that the Born scatterers of Hirschfeld have been shot down thoroughly by Mahmood et al recently in the context of the low superfluid density. Whatsoever, I find it a waste of my time to further attempt to

help the authors to see the light. It is a bit unfortunate that the paper is to a degree polluted by theoretical biasses, However, the data are interesting and surely worthy to be published in Nature communications.

We thank the referee for a very instructive discussion. We understand referee's criticism and we agree with his verdict that the data should be the most important outcome of this work. We think that our data do indeed speak for themselves. That is why we really tried to stay as far as possible from any theoretical overinterpretations and just state the obvious:

a) there is a coupling to something that looks like a boson.
b) energy of that excitation and coupling strength to it follow Tc
c) by comparing energies of know "bosonic" excitations, we eliminate some candidates and chose the one that fits the observed doping, temperature and momentum dependence of the observed renormalization.

We think that it is best to leave it to the readers to interpret these results as they deem appropriate.
